# Obstetrics and Gynecology

**Published:** 1895-09

**Authors:** 


### OBSTETRICS AND GYNECOLOGY.

Edited by Dr. Virgil O. Hardon.

### The Management of Labor Complicated by Heart Disease.

Barton Cook Hirst takes occasion to say, in the *American Journal of Obstetrics*, March, 1895, that the mortality, of some writers, of women in labor who are at the same time the subjects of organic heart disease, does not correspond with that of his own experience, it being entirely too high, and he lays his success entirely to his method of treatment, believing that his cases have been as serious as those of other men.

He instances as proof one woman with valvular disease (both insufficiency and stenosis of the left auriculo-ventricular orifice) who sat bolt upright in bed, day and night, for weeks before delivery, with labored breathing and face as blue as indigo; another with congenital heart disease of both mitral and tricuspid valves, a primipara at the age of forty-four, with advanced kidney disease to boot; a third with disease of the aortic orifice and an enormous aneurism of the arch of the aorta; a fourth with mitral disease of long standing, albuminuria, profound anemia, and an exceedingly rapid, weak pulse; and a number of other cases not so striking, of which he has unfortunately preserved no exact record.

In addition to the care every pregnant woman should have in the matter of diet, regulation of the bowels, exposure to cold and limitation of exercise, etc., he exhibits to those with heart disease iron and strychnia and one of the heart tonics, digitalis or strophanthus, in larger dose than would be used were she not pregnant.

The urine receives closer attention, and he terminates pregnancy prematurely, securing thereby an easy labor and avoiding the strain upon the heart that increases with every day in the last month of gestation.

When labor begins, digitalis and strychnia are administered in large doses hypodermatically until the os is the size of a dollar; then, in head-first labors, forceps are applied and the child extracted as rapidly as possible, without regard to the integrity of the maternal tissues and without anesthesia.

In some cases he has incised the cervix to facilitate delivery.

This he does to shorten labor and save the woman all the fatigue of voluntary muscular effort in the second stage and to insure hemorrhage from the lacerations, which he thinks the best safeguard against engorgement of the lungs and overstrain of the heart after childbirth.

Nitroglycerine and nitrite of amyl he keeps within easy reach in case of emergency.—*Ex.*

---

### TINCTURE OF IODINE IN POST-PARTUM HEMORRHAGE.

Dr. R. A. Brown (*Massachusetts Medical Journal,* February, 1895,) strongly urges the use of the tincture of iodine (one part to four of water) in the treatment of post-partum hemorrhage. He considers its power to excite contraction of the uterine tissues by reflex action its chief efficiency.

[The tincture of iodine is doubtless a valuable remedy in these cases, but we think it unwise to teach that it surpasses all others. Nothing will excite reflex action in the uterus more completely than the combined effect of one hand in the uterus (first making sure that the hand is thoroughly clean), and the other grasping tightly the fundus over the abdomen, as when expelling a placenta by Credé's method. The combined irritation of the walls of the womb from both within and without will immediately produce a strong contraction of its muscles and insure the closure of its sinuses. The clots should first be removed by the hand in the uterus. If hemorrhage is due to the rupture of an artery, a ligature or deep suture is of course called for.]—*Ex.*

---

### VAGINAL DOUCHES.

Kroenig's investigations lead him to the conclusion that the vaginal secretions during normal pregnancy are free from pathogenic germs and possess marked germicidal properties. Syringing the vagina with antiseptic solutions reduces or destroys this germicidal power, pure water slightly lessens it. He cannot verify the statements of Döderlein that certain signs mark a vaginal secretion, "a pathological secretion."

Kroenig concludes that prophylactic vaginal douches are not only useless but absolutely harmful, and should not be administered, even in cases in which gonorrheal infection is present.—*Ex.*

---

### PROPHYLACTIC MEASURES WHERE ECLAMPSIA IS THREATENED.

Dr. W. H. Haughey (*The Phys. and Surg.*, February, 1895,) in a paper on eclampsia, points out the value of eliminating the poison from the blood as quickly as possible through the three natural routes—the skin, bowels, and kidneys. If these are kept active he thinks we need have no fear of this occurring. When albumin is found in readily appreciable quantities, the time for active treatment has arrived.—*Ex.*

